# Formative Research to Identify Contributors to Risky Drinking and Adapt an Alcohol Reduction Intervention Among Young Adults Vulnerable to HIV: Qualitative Focus Group Study

**DOI:** 10.2196/76460

**Published:** 2026-02-26

**Authors:** Fidelis Sesenu, Sally Kirklewski, Bryce Takenaka, Jaime Brown, Erin Nicholson, Kimberly Haney, Kaylia Carroll, Arjee Restar, Donte Travon Boyd, Keisa Fallin-Bennett, Trace Kershaw, Carolyn Lauckner

**Affiliations:** 1 Center for Health, Engagement, and Transformation, University of Kentucky, 460 Healthy Kentucky Research Building, 760 Press Avenue, Lexington, KY, 40536, United States, 1 8595623335; 2 Department of Communication Studies, College of Communication and Information, University of Kentucky, Lexington, KY, United States; 3 Department of Social and Behavioral Sciences, Yale School of Public Health, Yale University, New Haven, CT, United States; 4 Department of Obstetrics and Gynecology, University of Kentucky, Lexington, KY, United States; 5 Markey Cancer Center, University of Kentucky, Lexington, KY, United States; 6 College of Social Work, The Ohio State University, Columbus, OH, United States; 7 Department of Family and Community Medicine, University of Kentucky, Lexington, KY, United States; 8 Charles Stewart Mott Department of Public Health, Michigan State University, Flint, MI, United States

**Keywords:** alcohol use, formative research, alcohol intervention, sexual minority, transgender, young adults, focus group

## Abstract

**Background:**

Across populations, risky drinking has been demonstrated to increase HIV risk behaviors. This is of special concern for sexually minoritized cisgender men and gender-diverse young adults (aged 18‐34 years), who report greater incidence of hazardous drinking (as defined by the Alcohol Use Disorders Identification Test - Consumption criteria) and HIV compared to their heterosexual and/or cisgender peers.

**Objective:**

This study aimed to examine alcohol perceptions, patterns of use, and the role that anti-LGBTQ+ (lesbian, gay, bisexual, transgender, queer) policies and discrimination played in alcohol risk behaviors for sexually minoritized cisgender men and gender-diverse individuals. Results were used to inform the development of an alcohol reduction intervention for this population.

**Method:**

A qualitative study was conducted with data collected via 4 focus groups among young adult sexually minoritized cisgender men and gender-diverse individuals in the United States from April to June 2023 (N=21). Participants were grouped according to identity as cisgender men, transgender men, transgender women, and nonbinary individuals. Transcripts were analyzed using codebook thematic analysis.

**Results:**

Alcohol use was described as a way to navigate belonging, social connection, and identity expression within LGBTQ+ contexts. Alcohol was viewed as a mainstay of LGBTQ+ spaces, with many using it as a social lubricant and coping mechanism for LGBTQ+-related stress, as well as for relaxation and having fun. Drinking intensity was often tied to an individual’s comfort with their evolving sexually minoritized cisgender men and gender-diverse identity, with drinking being higher in earlier stages of exploration. The consequences of drinking discussed by participants included impaired decision-making and negative effects on mental and physical health. Anti-LGBTQ+ laws and policies were seen as contributing to the further stigmatization of sexually minoritized cisgender men and gender-diverse individuals, and hazardous use of alcohol was used as a means of escape and coping.

**Conclusions:**

Alcohol use among sexually minoritized cisgender men and gender-diverse individuals is an important aspect of negotiating identity within different social settings and coping with stigma. Findings have valuable implications for tailoring alcohol reduction interventions for sexually minoritized cisgender men and gender-diverse young adults as they encounter stressors in real time.

## Introduction

The relationship between alcohol use and HIV risk is well defined, especially among sexually minoritized cisgender men and gender-diverse individuals, including transgender and nonbinary people [1-3]. Risky alcohol use, including binge drinking, is associated with condomless anal sex and sex while under the influence of other substances, which increases HIV and sexually transmitted infection risk [4-8]. Sexually minoritized cisgender men and gender-diverse individuals tend to have higher alcohol use than their heterosexual and/or cisgender peers [9-12]. Gender-diverse individuals, including transgender women, transgender men, and nonbinary individuals, are more likely to report hazardous drinking patterns, including both binge and heavy drinking, and they have a higher risk of alcohol use disorders compared to their cisgender peers [13-16]. Binge drinking is defined as consuming 5 or more drinks for males or 4 or more drinks for females in about 2 hours, while heavy drinking refers to having more than 15 drinks per week for males and more than 8 drinks per week for females [17,18]. Currently, there is limited guidance for defining binge or heavy drinking among gender-diverse individuals. Data from a nationally representative study by Kerridge et al [19] found that sexual minority adults have approximately 1.6-1.9 times greater odds of meeting criteria for past-year alcohol use disorder (AUD) and 1.7-2.4 times higher odds of lifetime AUD, relative to their heterosexual peers. Similarly, gender minority populations face increased risk. Azagba et al [13] found that gender nonconforming individuals had more than twice the odds of heavy drinking compared to cisgender women, while transgender men in particular reported 1.48 times higher odds of heavy drinking relative to cisgender women. Another large-scale study of health insurance claims found that transgender adults had a 2.75-fold higher prevalence of diagnosed AUD compared to cisgender adults [15]. Additionally, young adult sexually minoritized cisgender men and gender-diverse individuals are more likely to engage in risky alcohol use than older individuals [7].

Some of the strongest predictors of alcohol use in young adults are social norms and peer pressure, such as the expectation to drink heavily in social settings like parties, the belief that alcohol helps reduce social anxiety, or the pressure to conform to group behaviors in drinking games. These patterns appear to be consistent for sexually minoritized cisgender men and gender-diverse individuals [20-23], though one study found that sexually minoritized cisgender men and gender-diverse individuals were less influenced by their sexually minoritized peers to drink alcohol [24]. For some sexually minoritized cisgender men and gender-diverse young adults, alcohol plays a central role in personal identity, such as drinking to gain confidence to enter the queer social scene, drinking to meet gender norms, and drinking because queer events are often centered around alcohol [25-27]. One study found that sexually minoritized cisgender men and gender-diverse individuals tend to consume more alcohol around their sexually minoritized cisgender men and gender-diverse peers vs their cisgender heterosexual peers [28]. Elevated rates of anxiety and depression among sexually minoritized cisgender men and gender-diverse individuals have also been linked to increased alcohol use, with some research suggesting that these mental health challenges contribute to drinking as a form of self-regulation or coping [29]. Another study found that cisgender young adults had more positive motivations for drinking, such as celebrating and having a good time, while sexually minoritized cisgender men and gender-diverse young adults endorsed more negative reasons for drinking, such as drinking to decrease stress and interpersonal concerns [16].

In addition to the social aspects of alcohol use, location also plays a role in sexually minoritized cisgender men and gender-diverse individuals’ drinking behavior. Some sexually minoritized cisgender men and gender-diverse individuals seek out queer drinking spaces to reject gender norms related to drinking and feel more comfortable in the space [30]. Cisgender gay and bisexual men who report drinking across multiple types of settings (eg, their homes, friends’ homes, restaurants, and bars) are more likely to engage in heavy alcohol use and unprotected anal intercourse. Drinking across varied settings may provide more opportunities for alcohol consumption, increase exposure to different social and sexual environments, and reduce inhibitions or risk perception, thereby elevating the likelihood of risky sexual behavior [31]. This demonstrates the importance of considering locations in assessing risk behavior. There is limited research on the impact of drinking locations for transgender and nonbinary individuals.

In addition to the social aspects of alcohol consumption, stigma plays a significant role in influencing drinking behavior. The minority stress model [32] suggests that sexually minoritized cisgender men and gender-diverse individuals experience stigma, discrimination, and prejudice, which can create a stressful and unwelcoming social environment. This, in turn, contributes to negative mental health outcomes [32,33] and increased alcohol and substance use [21,34].

Beyond interpersonal stressors, the broader social and policy environments also shape drinking patterns among sexually minoritized cisgender men and gender-diverse individuals. Hatzenbuler and Link [35] define structural stigma as “societal-level conditions, cultural norms, and institutional policies that constrain the opportunities, resources, and well-being of the stigmatized.” Structural stigma, such as current state and federal legislation that restricts the rights of and access to health care in queer communities, leads to increased health inequities and negatively impacts mental health, as well as increased substance and alcohol use for sexually minoritized cisgender men and gender-diverse individuals [26,36-39]. Pachankis et al [40] found that experiencing past structural stigma was related to increased alcohol use. Restar and colleagues [41] also found that antitransgender policies were linked to increased harmful alcohol use and problematic substance use in transgender adults.

Understanding the motivations behind alcohol use, along with the social and physical contexts in which drinking occurs, is essential for developing effective interventions aimed at reducing alcohol use and associated sexual risk behaviors. Whether the goal is prevention or harm reduction, having an understanding of mechanisms, lay beliefs, and perceptions driving alcohol and sexual risk behaviors is essential for improving the effectiveness of interventions, especially those targeting marginalized and understudied populations. The goal of this formative study was therefore to examine alcohol perceptions, including anticipated benefits and perceived harms, patterns of use, and the role that anti-LGBTQ+ (lesbian, gay, bisexual, transgender, queer) policies and discrimination play in alcohol risk behaviors for sexually minoritized cisgender men and gender-diverse individuals in order to inform an alcohol reduction intervention adapted for this population. In this context, alcohol risk behaviors refer to patterns of use that increase the likelihood of negative outcomes, including binge drinking, heavy drinking, drinking to cope with stress or trauma, unprotected sex, and alcohol use in high-risk situations or environments with limited protective norms. Although national surveys have documented elevated alcohol use among sexually minoritized cisgender men and gender-diverse populations [2,11,13], there is limited research on the social and structural factors that shape alcohol use among sexually minoritized cisgender men and gender-diverse young adults. Much of the existing work relies on quantitative data, which often overlooks the lived experiences, interpersonal dynamics, and contextual influences that contribute to drinking behaviors. This study addresses that gap by using qualitative methods to explore contextually rich themes about how sexually minoritized cisgender men and gender-diverse young adults perceive and engage with alcohol.

## Methods

### Study Design

This study used a qualitative approach, with focus group discussions to gain a deeper understanding of alcohol perceptions, patterns of use, and the impact of anti-LGBTQ+policies and discrimination on alcohol risk behaviors among young adult sexually minoritized cisgender men and gender-diverse individuals. Focus groups are particularly useful in the quest to elicit and observe dialogue between participants, as well as gauge thematic consensus on issues [42,43].

### Participants and Setting

A total of 21 participants were recruited between April and June 2023 from the sexually minoritized cisgender men and gender-diverse community to participate in 4 focus groups. Participants were recruited using a combination of strategies, including flyer outreach in locations frequented by sexually minoritized cisgender men and gender-diverse individuals, such as LGBTQ+community centers, bars, and health clinics, referrals from other previous studies, social media recruitment, a dedicated study website, and institutional collaborations with academic and community partners. All participants were recruited in the United States. Inclusion criteria required participants to self-identify as a gender minority individual (eg, transgender man, transgender woman, or nonbinary person) or sexual minority man (ie, identifies as a man and with a sexual orientation other than heterosexual), be aged 18‐35 years, own a smartphone, self-report as HIV-negative, and screen positively for at-risk alcohol use. Individuals were considered to engage in at-risk alcohol use if they scored a 4 or higher on the Alcohol Use Disorders Identification Test - Consumption, a validated and widely used instrument for assessing harmful alcohol use [44].

This study focused specifically on sexually minoritized cisgender men and gender-diverse young adults because it was designed to inform the larger TRAC-ER (Tracking and Reducing Alcohol Consumption and Environmental Risk) alcohol intervention currently being tested through a randomized controlled trial with young adult sexually minoritized cisgender men and gender-diverse individuals [45]. While alcohol disparities among sexual minority women are well-documented, their exclusion reflects the intervention’s targeted scope on those most at risk for HIV rather than an assumption of lesser relevance. These criteria ensured alignment between the formative data and the intervention’s eligibility framework.

All focus group sessions were conducted virtually using the Zoom (Zoom Communications) platform. Focus groups were stratified according to sexual and gender identity groups (cisgender men, transgender men, transgender women, and nonbinary individuals) in order to increase participant comfort and obtain feedback about drinking culture specific to individuals’ communities. For focus group composition, if a participant selected multiple gender identities and one was a binary transgender identity (transgender man or transgender woman), the participant was scheduled into the corresponding transgender man or transgender woman group. If no binary transgender identity was selected (eg, nonbinary and genderqueer), the participant was scheduled into the nonbinary group. For descriptive statistics purposes only, participants who selected more than one gender identity are reported in a separate “multiple identities” category to avoid double-counting. Focus groups had an average of 5.3 (SD 2.2) participants per group. The target sample size was determined a priori based on feasibility, prior qualitative studies with similar populations, and the goal of reaching thematic saturation across diverse sexually minoritized cisgender men and gender-diverse identities. Saturation was assessed through ongoing analytic review of transcripts and was considered reached when no new themes or perspectives were observed in the final group discussions.

### Procedures

All consenting procedures were conducted remotely and electronically using the REDCap (Research Electronic Data Capture; Vanderbilt University) electronic consent framework. Eligibility was determined after completion of a screening survey and an additional video-based screening interview, which involved verbal confirmation of identity, consistency checks based on screener responses, and verification of contact information. If participants were determined to be eligible during the screening interview, the study coordinator reviewed the consent form with them and sent them the link to sign it electronically. The coordinator then scheduled focus group sessions based on participant availability and sent participants a brief demographics questionnaire to complete prior to the focus groups. Focus groups were conducted by 3 study team members who facilitated discussions, took notes, and managed the logistics of the Zoom session. Focus group sessions lasted 60‐90 minutes. Zoom audio recordings of sessions were saved, and all recordings were initially transcribed using Descript, a transcription software. Transcripts were then manually reviewed and corrected for accuracy by a research staff member (FS), with a secondary check conducted by another staff member (SK) to ensure reliability. Participants were deidentified using pseudonyms, which were used in all reporting of qualitative data.

### Focus Group Guide

Focus groups were conducted using a semistructured question guide (refer to Table 1 for a list of example questions). Questions were designed to address the general role of alcohol within the sexually minoritized cisgender men and gender-diverse community, alcohol use patterns, perceived benefits, negative consequences, and the impact of anti-LGBTQ+ policies, legislation, and discrimination on drinking behaviors. Question development was informed by a review of existing literature and refined through an iterative process with input from the study team over the course of 3 meetings held across a 4-week period.

**Table 1.** Summary of focus group guide.

Section	Example questions
General role of alcohol, perceptions of alcohol use, and alcohol use patterns	What role, if any, would you say that alcohol plays within the queer community? How does alcohol play a part in socializing within the queer community? How about your friend group? What types of situations do you typically associate with drinking? Probes: What places? Are these places more or less affirming? Are there certain situations or places that are uniquely associated with partying for the queer community?
Pros and cons of drinking	What do you see as the main benefits of drinking alcohol? Why do you or others like to do it? Probes: Any social effects? Any mental health effects? And what do you see as the cons or drawbacks of drinking? Probes: Social effects? Physical health effects? Effects on the use of other substances?
Influence of anti-LGBTQ+^a^ laws and policies on drinking behavior	With the rise in anti-LGBTQ+ laws and policies being debated and enacted, we are curious to hear how these could be affecting you. How have these laws and the rhetoric surrounding them impacted your life? Probes: How have they affected your mental health? How have these laws impacted your drinking behaviors? Probes: What about the drinking behaviors of your friends? The cisgender gay, transgender, nonbinary community at large? Are there certain members of the cisgender gay, transgender, nonbinary community who may be more likely to drink in response to these laws?
Conclusion	What do you think is the most important thing for us to include in our health promotion program to help reduce drinking within the queer community?

a LGBTQ+: lesbian, gay, bisexual, transgender, queer.

### Data Analysis

The analytic approach used in this study was codebook thematic analysis, where both deductive and inductive approaches are combined [46,47], aimed at ensuring the accuracy, reliability, and depth of thematic analysis. To begin, a codebook was developed, which served as the foundation for the coding process. This codebook was informed by both existing literature around drinking among sexually minoritized cisgender men and gender-diverse individuals [25,26,48,49] and an initial reading of the transcripts, allowing for a blend of deductive and inductive coding approaches. The inclusion of previous literature ensured that the coding was grounded in established knowledge, while the initial transcript reading allowed for the emergence of novel themes specific to the current data.

To enhance the credibility and trustworthiness of the analysis, we used several strategies recommended for qualitative research. Once the initial codebook was drafted, it underwent a review process by the entire study team, including the principal investigators (CL and TK), and was revised. Subsequently, 2 study team members (FS and SK) performed an initial independent coding of 2 transcripts and discussed coding decisions to establish consistency. Discrepancies were noted and resolved through consensus-based discussion first between the 2 coders and then among the wider research team to arrive at final code definitions. All 5 transcripts were then entered into Dedoose (SocioCultural Research Consultants LLC), a qualitative data analysis software, and coded by FS and SK using the revised codebook. Codes were first grouped into conceptually related clusters that captured shared ideas across participants. Through iterative discussion, the research team reviewed these clusters to identify broader patterns and overarching narratives. Themes were developed to reflect not only the content of participant responses but also the underlying meanings and social processes conveyed through their accounts. Finally, key themes and participant quotes from these data were then summarized and discussed by the study team during regular peer debriefing sessions to critically examine interpretations and ensure consistency with the data. Member checking was conducted with a subset of participants who later served as members of a Community Advisory Board (CAB). These individuals provided feedback on the thematic structure and interpretations. These procedures supported analytic rigor, transparency, and the dependability of the findings.

The research team included individuals with backgrounds in behavioral science, public health, psychology, and LGBTQ+ health research, some of whom identify as members of the LGBTQ+ community. The team acknowledged its shared investment in addressing health disparities and approached data collection and analysis with reflexivity regarding their own social positions, disciplinary training, and experiences. Regular team discussions were held to reflect on potential biases and to promote interpretive rigor throughout the coding and theme development process.

### Ethical Considerations

The study was approved by the Institutional Review Boards of the University of Kentucky and Yale University. All participants were aged 18 years or older and completed electronic informed consent prior to any study procedures. Participants received a US $40 electronic gift card upon completion of the focus group session. To protect confidentiality, all transcripts were deidentified by removing personal names and any potentially identifying details.

## Results

### Overview

Findings from the focus group discussions were organized into six broad themes: (1) navigating belonging and identity through reflections on alcohol in LGBTQ+ social contexts, (2) place-based drinking patterns in queer life, (3) anticipated benefits and reasons for drinking alcohol, (4) acknowledging the downsides of alcohol use, (5) individual and community impact of anti-LGBTQ+ discrimination on drinking behavior, and (6) alternative coping strategies for limiting alcohol consumption. Each of these broad themes is discussed in further detail below with its respective representative quotes.

### Sample Characteristics

As can be seen in Table 2, out of the 21 focus group discussion participants with an average age of 25.6 (SD 4.7) years, the largest group according to sexual orientation consisted of those who selected multiple sexual orientations (8/21, 38%), followed by gay (5/21, 24%). For gender identity, 8 out of 21 (38%) participants identified as nonbinary, gender nonconforming, or agender; 4 out of 21 (19%) as cisgender men; 4 out of 21 (19%) participants as transgender men; 1 out of 21 (19%) participants as transgender women; and 4 out of 21 (19%) participants selected multiple gender identities. Among those selecting multiple identities, 2 out of 4 (50%) participants included “trans woman,” and another 2 out of 4 (50%) included “trans man.” For focus group scheduling, participants with multiple identities that included a binary transgender identity were placed in the corresponding trans woman or trans man group, although they remain counted under “multiple identities” in Table 2 for descriptive clarity. Furthermore, the majority of participants had some college or higher education (19/21, 90%) and identified as non-Hispanic White (14/21, 67%). Finally, employment status and personal income varied, with 11 out of 21 (52%) participants being students or students who also worked and also earning less than US $15,000 annually.

**Table 2.** Demographic information for study participants (N=21).

Demographics characteristic	Values
Age (years), mean (SD)	25.6 (4.7)
Sexual orientation, n (%)	
Gay	5 (24)
Bisexual	2 (10)
Fluid	1 (5)
Pansexual	1 (5)
Queer	2 (10)
Lesbian	2 (10)
Multiple orientations	8 (38)
Gender identity, n (%)	
Cisgender man	4 (19)
Transgender man	4 (19)
Transgender woman	1 (5)
Nonbinary, gender nonconforming, or agender individual	8 (38)
Multiple identities	4 (19)
Hispanic or Latino origin, n (%)	
No	18 (86)
Yes, another Hispanic, Latino or Latina, or Spanish origin	1 (5)
Yes, Mexican, Mexican American, or Chicano or Chicana	2 (10)
Race, n (%)	
Black or African American	1 (5)
Multiracial	3 (14)
Non-Hispanic White	14 (67)
Asian	3 (14)
Education, n (%)	
Completed high school (graduate or GED^a^) - 12 years	1 (5)
Post–high school training other than college	1 (5)
Some college	8 (38)
College graduate	8 (38)
Postgraduate degree	3 (14)
Personal income (in US $), n (%)	
Less than 14,999	11 (52)
15,000-24,999	4 (19)
25,000-34,999	1 (5)
35,000-49,999	3 (14)
75,000-99,999	2 (10)
Employment status, n (%)	
Employed full-time (40+ h/wk)	5 (24)
Employed part-time (less than 40 h/wk)	3 (14)
Unemployed, not currently looking for work	1 (5)
Self-employed	1 (5)
Student	3 (14)
Student and working full- or part-time	8 (38)

a GED: General Educational Development certificate.

### Theme 1: Belongingness, Identity, and Alcohol in LGBTQ+ Social Contexts

#### Overview

Participants reflected on the roles that alcohol played in their communities and described alcohol as a meaningful part of their social and personal journeys, particularly within LGBTQ+ spaces. For many, alcohol consumption was an important part of sexually minoritized cisgender men and gender-diverse social settings; others reflected on its widespread visibility and consistent presence. Several participants also explored how alcohol use related to their evolving sense of identity, including using it as a temporary tool for self-acceptance or social ease and later distancing from it as they grew more confident in themselves. These narratives reveal how, for some sexually minoritized cisgender men and gender-diverse individuals, alcohol intersected with experiences of belonging, visibility, and identity formation.

#### Subtheme 1.1: Role of Alcohol in Social Settings

Among participants, alcohol’s role served a social function within the sexually minoritized cisgender men and gender-diverse community, acting as an icebreaker and facilitator for social interactions, and was often perceived as a necessity for engaging in community events. This sometimes led to the notion that drinking, drug use, and sexual activity were integral to the queer social fabric, as evidenced by the following quotations:

*If I’m looking for a place to, you know, feel safe and welcoming and, you know, have a good time with others in the community, it would be a gay bar, right? Or some sort of gay event where alcohol is, is present and prominent in the experience of the event.*

[Cisgender man]

*I’ll say for me, my primary interaction with queer people is at the bar. Like I go to a bar every Friday night. So, it’s centered around drinking.*

[Transgender an]

*If I’m with friends, I guess I found myself like drinking a little bit more ’cause like it’s just easy to be like, oh, do you wanna go get like a cheap beer and go play board games? So, I feel like in some ways drinking can be like a little inexpensive way of hanging out*.

[Cisgender man]

#### Subtheme 1.2: Observing the Popularity of Drinking in Queer Spaces

Several participants commented on the visible popularity and near-ubiquity of alcohol in queer social settings. They described drinking as a commonly encountered and widely accepted part of LGBTQ+ gatherings, events, and nightlife. These reflections highlighted how frequently alcohol was present, often as a default offering or unspoken feature of communal space, leading participants to reflect on the centrality of drinking to social rituals, leisure, and entertainment within their communities.

*I mean, it seems pretty popular. Again, I have a hard time speaking for just like the queer community in general. But I mean, it definitely seems to be pretty popular as far as everyone I talk to. Various substances, but yeah, alcohol is definitely one of them.*

[Transgender woman]

*As someone who is relatively new in my queerness, and you know, like what I see of the queer community is often revolved around alcohol, sex, or drugs. So, like my perception as someone who is trying to figure out my sexuality said, oh, these are the things that go along with it.*

[Cisgender man]

The second quote above illustrates not a judgment of the community, but a personal account of how their early impression of queer community life was tied to alcohol, as well as reflecting how cultural visibility of alcohol may shape emerging self-concepts.

#### Subtheme 1.3: Relationship Between Alcohol and Identity

The relationship between participants’ identities and their alcohol use was particularly salient. For some, alcohol initially served as a tool for fitting in or easing the discomfort of social scrutiny. One participant noted:

*I personally just like drink a lot just to like make myself feel like I fit in, just to feel like the other people’s eyes aren’t like prying at me.*

[Transgender man]

For others, alcohol facilitated the journey to self-acceptance, with its role diminishing as comfort with their identity grew.

*So, I, I think it helped me come out of the closet. And I think it’s, it’s just been a little evolution of my drinking habits. It’s been more of switching the mindset from like, I’m gonna get blasted drunk and stuff to more like, I’m just gonna use alcohol to relax, destress and that could be by myself and it doesn’t have to be binge drinking.*

[Cisgender man]

*I think as I’ve grown more used to and more comfortable with my sexuality, I don’t need alcohol to talk about these topics and to talk about my sexuality and to, to just express myself a little bit better. Alcohol definitely still helps. But it’s not like a necessary ingredient in the formula anymore, as much as, or as much as it was before.*

[Cisgender man]

### Theme 2: Place-Based Drinking Patterns in Queer Life

Participants noted the context of their physical and geographic environment as an important influence on drinking behaviors. In particular, participants felt queer bars played a significant role in alcohol consumption for sexually minoritized cisgender men and gender-diverse community individuals. One participant explained:

*I think in a lot of cities where there are queer spaces outside of like maybe an LGBTQ club center, most of the queer spaces are queer bars or queer clubs. So, there’s very few queer sober spaces I know.*

[Nonbinary individual]

However, this experience was not universal. For people who did not regularly patronize queer bars, they understated the role of alcohol in the community, as this participant highlighted:

*I think, funny enough, I find alcohol not really common in my experiences. I don’t really go to bars that much and I really don’t go to big gay bars that much.*

[Cisgender man]

This illustrates the diversity within the community, where the significance of alcohol varies widely depending on personal spatial habits and preferences.

In addition, participants also believed that locations played a critical role in shaping how individuals use alcohol, particularly as a tool for managing social anxiety in novel and/or unfamiliar spaces. As one participant put it:

*When I go to new spaces that I haven’t been to before, that’s when the anxiety is the absolute worst. I definitely will drink alcohol. Because it just really makes that anxiety go away and makes me not care.*

[Nonbinary individual]

Furthermore, some participants also described regularly drinking at home. This was often discussed within the context of being seen as a more affordable way to drink, or at least “pregame” before going drinking at bars. For others, drinking at home, either alone or while hanging out on online or virtual platforms like social networking websites with other queer people, was viewed as safer than drinking in public places.

*And those [drinking] spaces would always be either somebody’s house, somebody’s suite and then it would move on to maybe a frat house or like another person’s party.*

[Cisgender man]

*I don’t know if online [online or virtual platforms] is necessarily like a place to drink. But I mean, for me personally it is. If I want drinking socialization, I’m not gonna go to the bar. Not to mention the fact that like, I’m poor. So, it’s a waste of money. I can just spend $ 6 and get a bottle of Barefoot Moscato at Walmart and then just chug that through the night with my friends.*

[Transgender woman]

### Theme 3: Anticipated Benefits and Reasons for Drinking Alcohol

#### Overview

Participants expressed a multilayered relationship with alcohol, revealing its perceived benefits and the various motivations behind consumption. It was seen as a conduit for confidence, pleasure, coping, social connectivity, and a response to normative pressures. This complexity reflects both the enjoyment derived from drinking and its functional use as a tool for navigating the various dynamics of social life within the LGBTQ+ community.

#### Subtheme 3.1: Increases Confidence and Social Connectedness

For many, alcohol served as a confidence booster and social catalyst, lowering inhibitions and granting a level of boldness that can be difficult to muster in sober moments. The capacity of alcohol to diminish self-doubt, enhance personal openness, and provide liquid courage in social settings was encapsulated by the following participant comment:

*It’s a lot more comfortable to say some of those things that you’re coming to terms with and you’re not fully sure when you have one layer of your guard down, which alcohol can help you do.*

[Cisgender man]

Among participants, drinking was often perceived as an enabler of connections, lowering barriers to forming relationships. Bars were viewed as a common starting point for many relationships within the community, partly due to alcohol’s role in reducing social anxiety. Some participants admitted that certain levels of conversations and intimacy could also be facilitated by alcohol, which can lead to spontaneous and sometimes intense connections:

*Ask me if I will talk to somebody in a bar face-to-face that I find attractive or that I would love to go have a hook up with. No, hell no. I struggle with that now. Add a drink in my hand. Oh, we can have a conversation all night long. I will make out with you. We can go home together, we can cuddle, we can have sex.*

[Cisgender man]

Continuing on this theme, the feelings of not fitting neatly within conventional labels can make socializing daunting. Alcohol is then used as a social tool for inclusion:

*I don’t necessarily feel I fit in with many groups. Like as my label, I label myself as queer. I sometimes feel like I’m not gay enough to fit into the gay community. I’m not straight enough to fit into the straight community. I’m not bi enough. So, like for me, if I’m in a group of people, socially, there’s probably a drink in my hand just because I’m trying to fit in.*

[Cisgender man]

#### Subtheme 3.2: Enjoying the Act of Drinking

Participants also described a more intrinsic enjoyment of drinking, detached from its social functions. The simple pleasure of intoxication, especially in the company of friends, is highlighted as a desirable state. Moreover, the act of drinking can be a solitary form of indulgence and self-care, where a glass of wine accompanies a movie in a moment of personal relaxation:

*It’s like I enjoy beverages, like literally just the taste of them. And so I think for me it’s enjoyable to just actually drink alcohol, but then too, it, it is like something to do while you’re hanging out with people.*

[Nonbinary individual]

*I’m gonna put on a movie, have a couple of glasses of wine, and I’m just gonna vibe with myself.*

[Cisgender man]

For others, the ambiance of drinking settings contributed to its allure, turning a simple activity into an aesthetic statement:

*There’s kind of like a really fun aesthetic to drinking. My partner works at a bar. I like to go dress up when they’re working and just go and read at a bar. It’s not so much drinking, it’s just happens to be like, that’s the place I’m gonna be, but I wanna look cool doing it.*

[Cisgender man]

#### Subtheme 3.3: Social Norms and Social Influence

The normalization of drinking within certain social circles can create an environment where alcohol consumption is not just accepted but expected. The decision to drink can be influenced by the observation that “everyone else is doing it,” which can make the act seem both inconsequential and socially desirable. In discussions, social influence often emerged as a subtle yet powerful force that encouraged drinking behaviors. Participants in the focus groups recognized this social influence, with one individual stating:

*Sometimes you kind of think, well, everyone else is doing it too, at like parties and everything, so what’s the big deal?*

[Transgender man]

Another participant reflected on this normalization in relation to their identity, noting that it facilitated the initiation and continuation of their drinking habits:

*And I definitely do not personally associate it with my sexuality, but I know it’s normalized. So, it’s definitely has been easier for me to start drinking and keep drinking ’cause it’s normalized.*

[Cisgender man]

The role of social influence was also evident in the dynamics of flirtation and socializing, where the offer of a drink can serve the triumvirate role of invitation, validation, and obligation:

*But men want to buy me alcohol and so I really struggle to say no. Where like you’ll just be sitting and talking to somebody and they’ll be like, hey, do you want another one?… and it’s so easy to be like, yeah, sure, ’cause you want to flirt or you want to appear, I don’t know, fun and friendly. But then like my friend group, they’ll be like, oh, let’s do shots. Like, let’s do all this. And I’ve never been super rowdy, but there’s like a big pressure to, you know, sort of fit in or to like, get male attention, which is something I really want. Especially being a trans guy. Like, oh, I want those men to like me.*

[Transgender man]

#### Subtheme 3.4: Drinking to Cope With Anti-LGBTQ+–Related Stress

A common observation among participants was how the intersection of LGBTQ+ identity and societal pressures can lead to heightened levels of stress, for which alcohol becomes a coping mechanism. The desire to seek some reprieve and relief from the weight of this particular life stress through alcohol is shared among participants:

*Geez. I don’t know. It’s just life’s hard. Especially being queer. And for me and my friends, it’s at least a way to feel a little more free, a little more relaxed, a little more jovial when everything else is so terrible.*

[Transgender woman]

For those grappling with gender dysphoria or anxiety in romantic encounters, alcohol may also be used as a means to alleviate personal discomfort and affirm their identity:

*I have a lot of dysphoria still, and I haven’t had top surgery or anything like that. When I do meet someone, like if I meet someone on Grindr or on some kind of dating app. Prior to meeting up, I’m more likely to drink as well, just to kind of lower my own anxiety and try and get in that head space to where like, I’m, you know, I’m valid as a trans man and not just like trying to pretend*.

[Transgender man]

### Theme 4: Acknowledging the Downsides of Alcohol Use

#### Overview

While participants highlighted various perceived benefits of alcohol consumption, they also identified its potential drawbacks. The negative consequences of alcohol use spanned from immediate physical effects to more profound impacts on mental health, decision-making, and social dynamics. These reflections were often framed not in terms of addiction or dependence, but rather as moments of dissonance, recognizing when alcohol’s impact extended beyond its intended benefits.

#### Subtheme 4.1: Impaired Decision-Making

One of the more concerning aspects of alcohol use discussed by participants was its association with increased sexual risk-taking. Alcohol can impair judgment and lead to unprotected sex or forgetting to take medications like pre-exposure prophylaxis:

*Whether it be you forget to take your PrEP or you forget to use a condom or things like that, it can put you at a very high risk for contracting like STDs. It also puts you at a higher risk for like sexual assault, because it’s harder to emphasize no when, when you’re drinking.*

[Transgender man]

For some, the influence of alcohol on sexual decisions was a conscious trade-off, where the immediate gratification outweighed the potential risks:

*That is me. Hello! Alcohol leads me to make poor decisions for my sexual health. It’s important to point out, it’s not always that I forget. I just don’t want to or don’t care. It’s very like, nope, I can’t be bothered with this. Oh, I, there’s a park in my backyard. Let’s go walk around the park and see who’s around. Like, not always the best choices.*

[Transgender man]

Apart from sexual behaviors, alcohol was reported to impair decision-making in other significant ways. The influence of alcohol can extend to decisions that participants later regret, such as driving under the influence and making poor financial decisions:

*My family could not know the things I was doing at bars, so I was traveling across state lines to do a lot of that. That meant I was driving back either drunk or under the influence or however I got home.*

[Cisgender man]

*I feel like it’s so easy to like, you know, get drunk and keep buying more drinks. And then in the morning you’re kind of like, oh shit! Like half my credit card limit has been reached. Like, oh, I bought drinks for a ton of people last night. People I don’t even know.*

[Cisgender man]

*A con could be you might do something stupid that you might regret.*

[Transgender woman]

#### Subtheme 4.2: Negative Effects on Physical and Mental Health

In the discussions, the physical consequences of excessive drinking, such as hangovers and potential long-term health issues, were not overlooked. Participants were acutely aware of the immediate and long-term effects on their physical health, especially as they also got older:

*I know it has health risks and all that, but I mean, everything does.*

[Transgender woman]

*I’m at that point where I drink too much and then I feel like shit the next day. So that’s hard.*

[Transgender man]

Participants also expressed concerns about the exacerbation of negative emotions and mental health issues following alcohol consumption. Those with preexisting conditions like bipolar disorder also emphasized the particular risks of combining alcohol with their medications:

*I get very, very depressed and I’ll stay in my bed for a long time.*

[Nonbinary individual]

*It really affects me mentally as well because I have bipolar disorder and I’m on medication for that. So, mixing alcohol with those medications that you prescribe for your mental health can also affect you negatively.*

[Transgender man]

#### Subtheme 4.3: Negative Effects on Social Relationships

Excessive drinking was also identified as a factor that can strain social relationships, leading to situations where one might say or do things that harm their interpersonal, professional, and social connections:

*I’ve also had the next morning where I sober up and I’m like, what did I say last night? Should I really have said that?*

[Cisgender man]

*That comes with the hangover later on being like, why was I so aggressive out there? Why can’t I just be shy and cute? And then I would still have friends.*

[Nonbinary individual]

#### Subtheme 4.4: Effect of Alcohol Use on Other Substance Use

Participants acknowledged the risky connections between alcohol and other substance use. Participants spoke to the gateway nature of alcohol, where lowered inhibitions can lead to experimentation with and increased use of other substances:

*Like drinking opens a doorway to try other substances. If I’m drinking and someone says, hey, wanna try an edible? Sure! No, I shouldn’t probably. But the drinking, I’ve lowered it [inhibition], then the next day you’re dealing with trying to figure out what happened the night before.*

[Cisgender man]

*There’s a very fine slippery slope from, you know, having too much to drink and having it be much more easier to say yes to the drugs that have like, really, ruined my life in the past five years.*

[Cisgender man]

#### Subtheme 4.5: Creates False Confidence

Finally, the temporary confidence granted by alcohol was seen by some as artificial, with the recognition that reliance on substances like alcohol for self-assurance was unsustainable:

*I feel like it wasn’t good that sometimes I was using substances as a crutch to like give myself confidence. And I feel like I’ve gotten much better at being like, no, I don’t need to drink, I don’t need to smoke. Like, I should be able to dress myself and like be happy with what I’m wearing and like that should be it.*

[Cisgender man]

### Theme 5: Impact of Anti-LGBTQ+ Discrimination on Individuals’ Drinking Behavior

The experience of anti-LGBTQ+ discrimination cast a significant shadow over the lives of participants, with repercussions for alcohol use. Laws and the increasingly negative societal attitudes toward the LGBTQ+ community not only impacted participants’ mental health but also their drinking behavior, often leading to alcohol use as a form of escapism and maladaptive coping:

*I mean, it’s, it’s all terrible and I, I hate it and it just drives me to wanna drink even more to just not focus on it. Yeah. I hate that it’s even a political issue. It is just people existing.*

[Transgender woman]

*I definitely think that it has a huge effect on the queer population abusing substances more, not just using, but also abusing substances more because it’s an escape.*

[Transgender woman]

*I think definitely, at least for the people that I know and for me who are like, mostly nonbinary or, queer or like trans fem, alcohol has definitely been like a coping mechanism, unhealthy coping mechanism for just like the general climate of like, oh, like they wanna kill us kinda energy that’s going on in America and also just around the world.*

[Nonbinary individual]

From the discussions, social media appeared to influence perceptions of anti-LGBTQ+ discrimination and its ultimate impact on alcohol consumption. Social media amplified the effects of discrimination, where negative portrayals and hostile legislation frequently populated newsfeeds, contributing to stress and influencing drinking habits. This relationship was captured in the below participant quote:

*We actually played a game and everybody had to open up their social media apps on their phone and we had a bottle of tequila on the table. And we had to scroll through our social media and every time that we saw something, whether it be attacking transgender people, a shitty meme about transgender people or something about the bills passing, we had to take a shot. Let’s just say a party of seven trans men was trashed in less than 30 minutes ! It was just a lot to realize that like, it wasn’t just my timeline that was just flooded with all of these things.*

[Transgender man]

### Theme 6: Alternative Coping Strategies for Limiting Alcohol Consumption

In response to the normalization of alcohol within their social environments and its attendant risks, participants discussed strategies they used to moderate their drinking. These control mechanisms, ranging from self-imposed limits and self-monitoring of social media consumption to seeking social support, served as vital tools for managing their alcohol consumption.

Some individuals set explicit financial limits before engaging in social drinking, a practical strategy that naturally helped to cap their alcohol intake due to budget constraints. One participant explained their approach:

*Now, I’m like, all right, I’m going out to the bar. I might take 50 bucks. I might take a hundred bucks. That ain’t gonna get me drunk. Like the tolerance is high. So, I’m like, once that’s gone, if I’ve bought everybody a shot in the first round and I ain’t got no more money, probably going home, so I’m safe.*

[Cisgender man]

Awareness and education about the risks of excessive drinking also play a significant role in shaping participants’ drinking behaviors. Participants believed that by understanding the adverse effects of alcohol and the fact that extreme intoxication was not the norm, individuals were more inclined to drink responsibly:

*So, I think a lot of people when they’re in a community that drinks a lot, they think it’s very normal to get like blackout drunk and having that kind of information that like, hey, actually most people don’t do that.*

[Nonbinary individual]

Another less conventional but illustrative example of a strategy came from a participant who discussed the need to self-monitor their social media exposure. The participant framed this behavior as a way to manage emotional overwhelm and prevent spiraling into distress and stated they linked it to past drinking episodes. By minimizing exposure to triggering content, particularly related to anti-LGBTQ+ discrimination, the participant sought to maintain emotional stability without relying on alcohol:

*But I think the biggest issue, you know, is just having to constantly monitor my own consumption of social media so that I’m not sad all the time.*

[Transgender man]

The above findings across 6 thematic areas captured a range of experiences and insights that reflect the intricate relationship between alcohol consumption and the lived experiences of sexually minoritized cisgender men and gender-diverse individuals.

## Discussion

### Principal Findings

The conversations we had with our participants provided insight into the role alcohol plays in this sample of sexually minoritized cisgender men and gender-diverse individuals. We identified 6 themes that described how alcohol functions in sexually minoritized cisgender men and gender-diverse young adults’ lives, spanning community belonging, place-based patterns, anticipated benefits, negative effects, discrimination-related influences, and alternative coping strategies. Our participants discussed how drinking existed in various facets of their own lives as well as the larger LGBTQ+ community. Alcohol worked as both a way for individuals to build, explore, and express aspects of their identity and as a coping mechanism, while also having clear impacts on mental health, sexual behaviors, polysubstance use, and navigating social relationships. Finally, sexually minoritized cisgender men and gender-diverse individuals also contextualized how the increased prevalence of anti-LGBTQ+ policies and legislations has impacted how they move through the world, focusing particularly on what it means for their alcohol use behaviors.

Our findings have been consistent with other research related to alcohol use among sexually minoritized cisgender men and gender-diverse people. While overall there is limited research in this area, there are a few studies that have explored the ways in which alcohol operates as a boundary object for LGBTQ+ identity construction and placemaking [27,50,51]. For instance, Peralta [27] suggests that commercial gay scenes, such as bars and clubs, provide safe and affirming spaces that enable detachment from heteronormative hegemonies [27]. These social settings, similar to ones our participants discussed, play central roles in enabling communality and inclusivity within queer spaces [30]. As Hunt and colleagues [30] further discerned from their sample of LGBTQ+ youth, alcohol lowered inhibitions that allowed participants to feel free and less restricted from occupying their spaces, similar to the level of intimacy and depth our participants disclosed from drinking [30]. These findings provide insight into the existing gaps surrounding how alcohol use works toward sexual and gender identity formations of sexually minoritized cisgender men and gender-diverse people.

The pervasiveness of alcohol use and its place-based meaning were also highlighted by the sexually minoritized cisgender men and gender-diverse people in our study. According to critical geography and queer theory, queer spaces remain unfixed and spatially heterogeneous landscapes that are constructed within authoritative configurations of meaning [52-54]. Particularly, sexually minoritized cisgender men and gender-diverse young adults described how the nature of their alcohol use is dependent on the spaces they occupy. For instance, participants felt that gay or queer bars were places that felt “safe” and “welcoming” to socialize and drink. For individuals who do not frequent gay or queer bars, alcohol may be less meaningful. Additionally, sexually minoritized cisgender men and gender-diverse individuals who enter unfamiliar spaces rely on alcohol to ease social anxieties and increase their confidence to maneuver through them, which has been linked to forming relationships, having easier experiences with connecting with people, and finding joy through indulgence [25,55,56]. Thus, sexually minoritized cisgender men and gender-diverse spaces can be understood as a network of interconnected experiences, bringing together diverse ways of interacting with physical spaces, personal identity development, and social behaviors or practices [57]. For instance, a gay bar may serve as more than just a place to drink, as it acts as a space where sexually minoritized cisgender men and gender-diverse individuals can express their identities, connect with others, and challenge societal norms. An increased understanding of the place-based meaning of alcohol use has important implications for innovative alcohol reduction interventions that attend to the real-time and nuanced characteristics of sexually minoritized cisgender men and gender-diverse spaces. Particularly for alcohol reduction interventions, understanding the contextual meanings of locations that potentially influence sexually minoritized cisgender men and gender-diverse alcohol use will help enable future researchers to mobilize more tailored prevention and harm reduction interventions.

Despite the reported benefits and the meaning that alcohol use has within LGBTQ+ spaces, many of the sexually minoritized cisgender men and gender-diverse participants discussed drinking in relation to coping against larger structural forces, such as anti-LGBTQ+ discrimination, stigma, and heterosexism and cissexism. This was unsurprising given that sexually minoritized cisgender men and gender-diverse individuals, who experience greater hostility and stress, tend to cope by engaging in greater alcohol use [26,27,58,59]. To our knowledge, this is one of the few qualitative studies that discusses specific alcohol-related implications at the intersection of sexually minoritized cisgender men and gender-diverse identities, place, and anti-LGBTQ+ systems and structures of oppression. Participants’ narratives described in this study related to the escalation of anti-LGBTQ+ rhetoric have been previously hypothesized to shape a greater inclination toward alcohol use and binge-drinking behaviors, as well as exacerbate other negative consequences related to mental health and well-being and sexual health [40,49,60]. However, future research, both qualitative and quantitative, should explore how anti-LGBTQ+ policies reconfigure queer spaces and sexually minoritized cisgender men and gender-diverse individuals’ negotiation of alcohol use. In particular, future research may benefit from identifying intervention components or content that can disrupt the linkage between adverse mental health stressors and alcohol and substance use, as well as how these outcomes may be buffered via training on healthy coping strategies and harm reduction approaches.

### Limitations

While our study possesses various strengths, it is not without limitations. The responses discussed here were provided as one part of a larger focus group to obtain feedback on an alcohol reduction program and its app-messaging materials. Thus, this may have limited the extent to which we were able to engage all participants for each aspect of the discussion. Social desirability and potential recall bias may have informed how participants responded, given that these conversations involved sensitive topics involving alcohol use, as well as other experiences related to substance use and sexual health. Participants were largely conveniently sampled through universities and proximal community organizations. As such, the transferability of findings to other sexually minoritized cisgender men and gender-diverse people located in different areas in the United States may be limited.

### Study Implications

This study poses important implications for research and interventions for alcohol use. Our findings suggest that alcohol reduction interventions can benefit from discussions around queer-affirming and place-based technologies. They also underscore the importance of acknowledging the perceived benefits of drinking and the role alcohol plays in fostering community among sexually minoritized cisgender men and gender-diverse young adults. These insights highlight the value of harm reduction approaches, because harm reduction offers a safe, flexible, and effective framework for supporting individuals in cultivating healthier relationships with alcohol.

Furthermore, insights from this study lead to broader lessons for tailoring alcohol interventions to sexually minoritized cisgender men and gender-diverse populations. First, intervention strategies should be adapted to reflect the diversity of motivations for drinking, as well as to address the negative effects of harmful alcohol use among target populations. Second, identity-specific stressors, such as anti-LGBTQ+ discrimination and place-based stressors, should be explicitly addressed when identifying drinking triggers and developing coping strategies. Third, the incorporation of harm reduction principles is essential, in acknowledgment of the central role that alcohol plays in the social lives of many sexually minoritized cisgender men and gender-diverse individuals, making complete abstinence unlikely or undesirable. Fourth, interventions can be strengthened by using customizable, real-time technologies, such as smartphone apps, to deliver personalized prevention messages that are both context-sensitive and user-generated. We leveraged these broader lessons to inform the adaptation of the manualized TRAC-ER alcohol intervention, originally developed for people with HIV and now being tested through a randomized controlled trial with young adult sexually minoritized cisgender men and gender-diverse individuals [45]. While the intervention is still undergoing testing, the formative insights presented here supported its alignment with participants’ lived experiences and offer scalable implications for future alcohol reduction interventions targeting sexually minoritized cisgender men and gender-diverse populations.

Building on the broader lessons above, several themes informed specific TRAC-ER adaptations. Themes 1, 3, and 4 (belonging and identity in LGBTQ+ contexts; anticipated benefits and reasons for drinking; downsides of alcohol use) led us to revise the counseling sessions that focus on developing change plans and identifying triggers so that content now reflects the diversity of motivations for drinking (social connection, coping, and relaxation) and explicitly addresses negative effects discussed by participants. Themes 2 and 5 (place-based patterns and impact of anti-LGBTQ+ discrimination) informed updates to the triggers session to include identity-specific stressors (eg, anti-LGBTQ+ discrimination) and place-based stressors, along with planning prompts and messaging to support safer choices in bars, clubs, and home settings. Themes 4 and 6 (downsides of alcohol use; alternative coping strategies) motivated the addition of a harm-reduction session that supports nonabstinence goals (eg, pacing and spending caps) and strengthens self-monitoring and peer support. Finally, across themes, we added a smartphone feature that allows participants to author their own prevention and harm-reduction messages tailored to personal triggers and current location, delivered in real time (user-generated, context-sensitive support).

From a policy perspective, reducing alcohol-related harm among sexually minoritized cisgender men and gender-diverse young adults requires addressing structural stigma that drives stress-related drinking. At the population level, protections that safeguard gender-affirming care, antidiscrimination enforcement in employment and housing, and safe public accommodations may reduce stress-induced alcohol use. In practice, harm-reduction–oriented interventions should include goal-setting beyond abstinence (eg, drink counting, pacing, and low-risk drinking plans), trigger mapping for identity-specific and place-based stressors, and linkage to culturally competent mental health and social support. Embedding these elements in LGBTQ+-affirming community spaces and digital tools may improve uptake and equity.

### Conclusion

Alcohol use among sexually minoritized cisgender men and gender-diverse young adults is intertwined with identity, social context, and exposure to structural stigma. Interventions should be adapted to the diversity of motivations for drinking, including social connection, coping, and mood regulation, while directly addressing the negative effects of harmful use. Pairing harm-reduction strategies with attention to identity-specific and place-based triggers, as well as policies prioritizing protections that lessen stigma-related stress, can better align support with lived experience. Finally, interventions can be strengthened through customizable, new areas of implementation that use mobile technology to support sexually minoritized cisgender men and gender-diverse individuals by delivering personalized, context-sensitive, user-generated prevention and/or harm-reduction messages in the settings where decisions are made. Future work should evaluate these design principles at scale and alongside policies that reduce stigma-related stressors.

## Abbreviations

AUD: alcohol use disorder
CAB: Community Advisory Board
LGBTQ+: lesbian, gay, bisexual, transgender, queer
REDCap: Research Electronic Data Capture
TRAC-ER: Tracking and Reducing Alcohol Consumption and Environmental Risk
